# Modeling transcranial magnetic stimulation coil with magnetic cores

**DOI:** 10.1088/1741-2552/acae0d

**Published:** 2023-01-25

**Authors:** Sergey N Makaroff, Hieu Nguyen, Qinglei Meng, Hanbing Lu, Aapo R Nummenmaa, Zhi-De Deng

**Affiliations:** 1 Department of Electrical & Computer Engineering, Worcester Polytechnic Institute, Worcester, MA, United States of America; 2 Magnetic Resonance Imaging and Spectroscopy Section, Neuroimaging Research Branch, National Institute of Drug Abuse Intramural Research Program, Baltimore, MD, United States of America; 3 Athinoula A. Martinos Center for Biomedical Imaging, Massachusetts General Hospital, Harvard Medical School, Boston, MA, United States of America; 4 Computational Neurostimulation Research Program, Noninvasive Neuromodulation Unit, Experimental Therapeutics & Pathophysiology Branch, National Institute of Mental Health Intramural Research Program, National Institutes of Health, Bethesda, MD, United States of America

**Keywords:** transcranial magnetic stimulation (TMS), coil design, nonlinear magnetic core, *B*–*H* magnetization curve, anhysteretic *B*–*H* curve, boundary element fast multipole method (BEM-FMM), numerical modeling

## Abstract

*Objective.* Accurate modeling of transcranial magnetic stimulation (TMS) coils with the magnetic core is largely an open problem since commercial (quasi) magnetostatic solvers do not output specific field characteristics (e.g. induced electric field) and have difficulties when incorporating realistic head models. Many open-source TMS softwares do not include magnetic cores into consideration. This present study reports an algorithm for modeling TMS coils with a (nonlinear) magnetic core and validates the algorithm through comparison with finite-element method simulations and experiments. *Approach.* The algorithm uses the boundary element fast multipole method applied to all facets of a tetrahedral core mesh for a single-state solution and the successive substitution method for nonlinear convergence of the subsequent core states. The algorithm also outputs coil inductances, with or without magnetic cores. The coil–core combination is solved only once i.e. before incorporating the head model. The resulting primary TMS electric field is proportional to the total vector potential in the quasistatic approximation; it therefore also employs the precomputed core magnetization. *Main results.* The solver demonstrates excellent convergence for typical TMS field strengths and for analytical *B*–*H* approximations of experimental magnetization curves such as Froelich’s equation or an arctangent equation. Typical execution times are 1–3 min on a common multicore workstation. For a simple test case of a cylindrical core within a one-turn coil, our solver computed the small-signal inductance nearly identical to that from ANSYS Maxwell. For a multiturn rodent TMS coil with a core, the modeled inductance matched the experimental measured value to within 5%. *Significance.* Incorporating magnetic core in TMS coil design has advantages of field shaping and energy efficiency. Our software package can facilitate model-informed design of more efficiency TMS systems and guide selection of core material. These models can also inform dosing with existing clinical TMS systems that use magnetic cores.

## 1. Introduction

It has long been known that soft magnetizable materials [1] can be used as a core of transcranial magnetic stimulation (TMS) coils [2, 3], (see also review [4]). A magnetic core may focus or redirect both the coil magnetic and electric fields to a region of interest and thus can increase the energy efficiency of TMS considerably. The use of a magnetic core can potentially offer an efficient way to produce stimulation fields using a smaller device that requires less energy and produces less heating [5].

Since the TMS magnetic field strengths typically reach 1.5 Tesla (T) or higher, it is desirable to use materials that saturate at or above 1.5 T. One suitable material, for example, is vanadium permendur. Other suitable materials include the metallic glasses (i.e. Metglas), permalloy, supermalloy, powdered iron, and silicon irons or silicon steels, in particular, 3% grain oriented steel (magnesil). Ferrite could also be used, although it is not preferred, due to the fact that it saturates at 0.5 T [2, 3]. New magnetic materials suitable for high-frequency, high-power applications in modern switching power electronics (transformers, pulse power cores, high-frequency inductors) are actively being developed [6–9]. These materials might potentially become very suitable candidates for modern and future medical TMS applications.

Despite their desirable properties, TMS magnetic cores are not widely used at present. Neuronetics, Inc. of PA, USA is the only company that has been widely (and successfully) employing the magnetic (iron) core technology for TMS applications [5]. The modern research on TMS coils with the magnetic cores is also rather sparse [4, 10–16]. There seem to be several potential questions pertinent to the subject matter. First, the time constant of a simple RL circuit modeling a coil is $L/R$ where *L* is coil’s inductance and *R* is coil’s resistance. Adding the magnetic core might significantly increase the inductance hereby increasing the duration of the TMS pulse and decreasing the induced electric field following the Faraday’s law of induction. It is however unclear whether or not the inductance really changes that much, for all possible geometries and especially in saturation. There are some optimistic examples in the literature [10]. Numerical modeling could help us to investigate this problem.

Second, there is doubt that the addition of ferromagnetic cores is practical as they might enter magnetic saturation in the field range needed for TMS [17]. As long as the material is (nearly) saturated, its major advantage—the much higher permeability—may largely be lost. It is however unclear whether or not the remaining permeability increase might still be sufficiently high, especially for the new magnetic materials. Numerical modeling can help us answer this question.

Third, Koponen and colleague noted that although ‘an iron core can increase the energy efficiency of a TMS coil considerably; however, this increase comes at a cost of increased bulkiness…’ [18]. This is indeed true, but it is not entirely clear how bulky should the core really be in different situations. Numerical modeling could again help us to quantify the necessary core volume in every specific case.

The magnetic core modeling is a complicated non-linear problem. The finite element method (FEM) and the finite difference method are the major tools of modeling TMS coils with magnetic cores [10–13]. FEM has been extensively developed and used in more general power-electronics problems with the magnetic cores including quite sophisticated hysteresis and anisotropic models [19–25].

An industry-leading commercial FEM software package, ANSYS Maxwell, could be employed to model the coil with the core in the magnetostatic approximation including arbitrary anhysteretic *B*–*H* curves, non-zero coercivity, self- and mutual inductances, etc. Though the ANSYS Maxwell Eddy current solver could output the induced electric field directly, the corresponding solution appears highly oscillatory and, in the authors’ own experience, might be less accurate. A very relevant demonstration is given in [11]—see figure 7, and especially figures  8 and 11 of this reference. Furthermore, both these software packages tend to slow down when a realistic high-resolution head model is included into consideration. On the other hand, the excellent open-source TMS software SimNIBS [26–28], can incorporate anatomically-accurate head models, but it cannot yet model coil inductance or a coil with a magnetic core. Therefore, there is a need for a tool that could model the coil, the core, and the head in one package and be appropriate for solving the specific TMS tasks and testing different existing and prospective cores.

In this study, we aim to develop and disseminate such a tool. We expand the previously developed boundary element fast multipole method (BEM-FMM) engine [29, 30] to a rapid modeling of TMS coils with a linear and nonlinear core. Note that the accurate modeling of the linear core problems via the BEM-FMM appears mostly straightforward and is fast. It is accomplished using the duality between magnetostatic, electrostatic, and DC BEM conduction analyses [31] since the conduction BEM-FMM solver is already available [30, 32, 33].

For the nonlinear (and/or anisotropic) analysis, we have to introduce a volumetric tetrahedral mesh in addition to the standard BEM triangular surface mesh. Then, the problem is how to expand the standard fast linear-core BEM formulation [34–36] to the nonlinear case. One possible solution is based on the introduction of volume magnetic charges and using the method of double integral equation (surface+volume) [37]. However, it might be too complicated. In this study, we suggest using a different simpler method that still utilizes only one integral equation of the standard BEM. At the same time, it applies this equation to all faces of the underlying tetrahedral mesh of the core, including not only the boundary faces but also all inner faces. In other words, we assume a constant permeability within each tetrahedron and impress the surface charge density at each outer and inner face to satisfy the boundary condition of the continuous normal component of the magnetic flux density.

In the following sections, we will first present the methods for modeling TMS coils with and without magnetic core, numerical implementation of the solver, and material models for magnetic cores. Next, we will compare solutions generated by our software with those from a commercial FEM solver. We will further validate our software by comparing the model to experimental measurements of a constructed TMS coil, as well as to analytic solution (the latter is presented in the supplementary material).

## 2. Methods

### 2.1. Solving the primary field of TMS coil without magnetic core

The primary field of a TMS metal coil and the primary coil current are assumed to be unaffected by the magnetic core. The primary field of a TMS coil will be denoted by superscript p. The static coil is characterized by a conduction current density $\mathbf{j}(\mathbf{r})$ (A m^−2^) everywhere within metal conductors. The magnetic vector potential generated by coil currents flowing within all metal windings with volume *V* is found from Ampère’s law

\begin{equation*} \mathbf{A}^\textrm{p}(\mathbf{r}) = \frac{\mu_0}{4\pi}\int_V \frac{\mathbf{j}(\mathbf{r}^{^{\prime}})}{\left|\mathbf{r}-\mathbf{r}^{^{\prime}}\right|} \mathrm{d}\mathbf{r}^{^{\prime}}, \end{equation*}

where *µ*_0_ is magnetic permeability of vacuum (air). In the quasistatic approximation, the separation of variables applies. It yields $\mathbf{j}(\mathbf{r},t) = I(t)\mathbf{j}(\mathbf{r})$, where *I*(*t*) is the temporal waveform of a TMS pulse generator and $\mathbf{j}(\mathbf{r})$ is the static current distribution map. The induced primary electric field, $\mathbf{E}^\textrm{p}(\mathbf{r},t) = -\partial\mathbf{A}^\textrm{p}(\mathbf{r},t)/\partial t$, therefore becomes

\begin{equation*} \mathbf{E}^\textrm{p}(\mathbf{r},t) = -\frac{\partial I(t)}{\partial t}\mathbf{A}^\textrm{p}(\mathbf{r}), \end{equation*}

at any time moment. Similarly, the static part (this term will be omitted in what follows) of the magnetic flux density is found from Biot–Savart law in the form:

\begin{align*} \mathbf{B}^\textrm{p}(\mathbf{r}) &amp;= \nabla\times\mathbf{A}^\textrm{p}(\mathbf{r}) \nonumber \\ &amp;= \frac{\mu_0}{4\pi} \int_V \nabla\times \frac{\mathbf{j}(\mathbf{r}^{^{\prime}})}{\left|\mathbf{r}-\mathbf{r}^{^{\prime}}\right|} \mathrm{d}\mathbf{r}^{^{\prime}}\nonumber \\ &amp; = \frac{\mu_0}{4\pi} \int_V \frac{\mathbf{j}(\mathbf{r}^{^{\prime}})\times (\mathbf{r}-\mathbf{r}^{^{\prime}})}{\left|\mathbf{r}-\mathbf{r}^{^{\prime}}\right|^3} \mathrm{d}\mathbf{r}^{^{\prime}}, \end{align*}

and the magnetic field intensity in free space is finally given by

\begin{equation*} \mathbf{H}^\textrm{p}(\mathbf{r})= \frac{1}{\mu_0}\mathbf{B}^\textrm{p}(\mathbf{r}).\end{equation*}

### 2.2. Solving secondary field with magnetic core

The magnetic core is characterized by its relative permeability, *µ*_r_, which may be field independent (a linear core) or change with the field intensity (a nonlinear core). In the presence of the magnetic field of the coil—the primary field given by equations (1)—the core material becomes *magnetized*; that is the microscopic dipoles acquire a net alignment along certain direction(s). Their net effect results in a secondary magnetic vector potential $\mathbf{A}^\textrm{s}$, secondary magnetic flux $\mathbf{B}^\textrm{s}$, and the secondary magnetic field $\mathbf{H}^\textrm{s}$—the response of the magnetic materials. The total potential, flux, and the field become the sum of both the primary and the secondary fields. The magnetic vector potential of a single magnetic dipole with moment $\mathbf{m}(\mathbf{r}^{^{\prime}})$ located at $\mathbf{r}^{^{\prime}}$, is given by

\begin{equation*} \mathbf{A}^\textrm{s}(\mathbf{r})=\frac{\mu_0}{4\pi}\frac{\mathbf{m}(\mathbf{r}^{^{\prime}})\times(\mathbf{r}-\mathbf{r}^{^{\prime}})}{\left|\mathbf{r}-\mathbf{r}^{^{\prime}}\right|^3}. \end{equation*}

In the magnetized object, each volume element $\mathrm{d}\mathbf{r}^{^{\prime}}$ carries a dipole moment $\mathbf{m} = \mathbf{M}(\mathbf{r}^{^{\prime}})\mathrm{d}\mathbf{r}^{^{\prime}}$. Here, **M** is magnetization or dipole moment per unit volume. It is expressed through the magnetic field within the magnetized object in the form

\begin{align*} \mathbf{M}(\mathbf{r}) &amp;= (\mu_\textrm{r}-1)\mathbf{H}(\mathbf{r}), \nonumber\\ \mathbf{H} &amp;= \frac{1}{\mu_0}\mathbf{B}-\mathbf{M}, \nonumber \\ \nabla\cdot\mathbf{B} &amp; = 0, \end{align*}

where *µ*_r_ is the relative permeability of the core. Therefore, the secondary magnetic vector potential generated by the magnetized core with the volume *W* becomes (see, for example, [34])

\begin{equation*} \mathbf{A}^\textrm{s}(\mathbf{r}) = \frac{\mu_0}{4\pi}\int_W \frac{\mathbf{M}(\mathbf{r}^{^{\prime}})\times(\mathbf{r}-\mathbf{r}^{^{\prime}})}{\left|\mathbf{r}-\mathbf{r}^{^{\prime}}\right|^3} \mathrm{d}\mathbf{r}^{^{\prime}}, \end{equation*}

anywhere in space. Once $\mathbf{H}(\mathbf{r})$ is known, $\mathbf{A}^\textrm{s}(\mathbf{r})$ can be found from equations (2*b*) and (2*c*). However, $\mathbf{H}(\mathbf{r}) = \mathbf{H}^\textrm{p}(\mathbf{r})+ \mathbf{H}^\textrm{s}(\mathbf{r})$ is now the total field. The secondary field $\mathbf{H}^\textrm{s}$ present in this expression is not known. So is the secondary flux $\mathbf{B}^\textrm{s} = \mu\mathbf{H}^\textrm{s}$. The goal of the numerical modeling is to solve for them.

#### 2.2.1. Solving linear core

In the magnetoquasistatic approximation, the displacement current is negligible. Thus, the approximate form of Ampère’s law is the continuity condition of the current density, **j**. Anywhere within the coil current-free region, $\mathbf{j} = 0$. Therefore, Ampère’s law for the secondary field yields $\nabla\times\mathbf{H}^\textrm{s}(\mathbf{r}) = 0$. Therefore, the secondary field can be written in the form of a gradient of a certain function—the (full or reduced) magnetic scalar potential with the units of ampere (A) [34, 36]. Bypassing operations with the potential, one could choose a representation in the form of magnetic charges—the surface charge density $\rho_\textrm{s}(\mathbf{r})$ with the units of weber per square meter (Tesla). The conceptual magnetic charges are a useful abstraction that aid in the solutions of (nonlinear) magnetostatics [34, 38]. Namely, the magnetic material can be removed and replaced, in the evaluation of the secondary field $\mathbf{H}^\textrm{s}(\mathbf{r})$ and other secondary quantities, by the surface bound charges so that one has [34]

\begin{equation*} \mathbf{H}^\textrm{s}(\mathbf{r})=-\frac{1}{4\pi\mu_0} \nabla \int_S \frac{\rho_\textrm{s}(\mathbf{r}^{^{\prime}})}{\left|\mathbf{r}-\mathbf{r}^{^{\prime}}\right|} \mathrm{d}\mathbf{r}^{^{\prime}}.\end{equation*}

Equation (3*a*) is identical with the corresponding electrostatic charge representation (after replacing permeability by permittivity and **H** by **E**) or with the DC current representation (after replacing permeability by conductivity and **H** by **E**). Other definitions of magnetic charges are possible, to within a constant factor *µ*_0_ [34]. It can be shown [34] that $\rho_\textrm{s} = \mu_0 \mathbf{n}\cdot \mathbf{M}$ where **n** is the outer normal to the core surface *S*. Otherwise, the divergence of magnetization is equal to zero.

Equation (3*a*) is augmented with the boundary condition on surface *S* with the local normal vector $\mathbf{n}(\mathbf{r})$ and with *µ*, *µ*_0_ being the permeabilities inside and outside with regard to the direction of its outer normal vector, respectively,

\begin{equation*} \mu \mathbf{n}(\mathbf{r})\cdot \mathbf{H}_\textrm{in}(\mathbf{r})=\mu_0 \mathbf{n}(\mathbf{r})\cdot \mathbf{H}_\textrm{out}(\mathbf{r}), \quad\mathbf{r}\in S \end{equation*}

where $\mathbf{H}_\textrm{in/out}$ is the *total* magnetic field just inside/outside the permeability interface. After substitution of equation (3*a*) into equation (3*b*) and using the principal value of the singular surface integral [39], an integral equation for *ρ*_s_—the Fredholm equation of the second kind referred to in [34] as a Phillips-type equation—is obtained in the form:

\begin{align*} &amp;\frac{\rho_s(\mathbf{r})}{2}-K\mathbf{n}(\mathbf{r}) \int_S \frac{1}{4\pi}\frac{\mathbf{r}-\mathbf{r}^{^{\prime}}}{\left|\mathbf{r}-\mathbf{r}^{^{\prime}}\right|^3} \rho_s(\mathbf{r}^{^{\prime}}) \mathrm{d}\mathbf{r}^{^{\prime}}\nonumber\\ &amp;\quad= K\mathbf{n}(\mathbf{r})\cdot \mathbf{B}^\textrm{p}(\mathbf{r}), \quad \mathbf{r}\in S \end{align*}

where the magnetic permeability contrast $K = (\mu-\mu_0)/(\mu+\mu_0)$ is uniquely defined at the material interface.

Equation (3*c*) coincides with the corresponding result for quasi-static conduction problems in TMS, transcranial electrical stimulation (TES), and electro-/magneto-encephalography (EEG/MEG) modeling [30, 32, 33] when the substitution stated above is made. It is solved exactly in the same way, using the generalized minimum residual method (GMRES [40, 41]) for the iterative solution and the fast multipole accelerator [42, 43], for computing the matrix vector product. The GMRES convergence is excellent [30, 32, 33]. For large $\mu\gg\mu_0$, the subtraction approach is used to correct a numerical error inside the core [31, 44]. After $\rho_\textrm{s}(\mathbf{r})$ is found, $\mathbf{H}^\textrm{s}(\mathbf{r})$ is computed from equation (3*a*), and $\mathbf{A}^\textrm{s}(\mathbf{r})$ is computed from equation (2*c*). Thus, our method is as follows:

(a) Given the known $\mathbf{B}^\textrm{p}$ of the coil in free space and the known *µ* of the core, solve the integral equation (3*c*) for the density of effective surface magnetic charge $\rho_s(\mathbf{r})$ residing on the core surface, via the FMM.
(b) Find the secondary field of the core, $\mathbf{H}^\textrm{s}(\mathbf{r})$, from equation (3*a*). Then, find the total field $\mathbf{H}(\mathbf{r})$ and magnetization $\mathbf{M}(\mathbf{r})$ from equation (2*b*).
(c) Substitute $\mathbf{M}(\mathbf{r})$ in equation (2*c*) and find the secondary magnetic vector potential $\mathbf{A}^\textrm{s}(\mathbf{r})$.
(d) Finally, add $\mathbf{A}^\textrm{s}(\mathbf{r})$ to the potential of the coil in free space, $\mathbf{A}^\textrm{p}(\mathbf{r})$, and obtain the total magnetic vector potential, $\mathbf{A}(\mathbf{r})$, of the coil with the core.

#### 2.2.2. Solving nonlinear core

When the core permeability becomes field-dependent, volume magnetic charges can be introduced along with the surface charges [34]. One more integral equation thus has to be added to equation (3*c*) and the coupled equations have to be solved simultaneously. This approach was in particular described and tested in an excellent study from [37]. However, it might be too complicated. In this study, we suggest using a different method that still utilizes only *one* integral equation (3*c*). At the same time, it expands it to all faces of an underlying tetrahedral mesh of the core, including not only the boundary faces as in equation (3*c*) but also all inner faces. Our method is as follows:

(a) A normal vector $\mathbf{n}(\mathbf{r})$ is introduced for any inner face based on two adjacent tetrahedra. It is directed from a tetrahedron ‘plus’ to a tetrahedron ‘minus’. Their initial choice is arbitrary. The initial local permeability $\mu(|\mathbf{H}^\textrm{p}|)$ is assigned to each tetrahedron at the first step since $\mathbf{H}^\textrm{p}$ is known as the coil field in free space.
(b) Secondary magnetic field $\mathbf{H}^\textrm{s}(\mathbf{r})$ is assumed to be constant within every small tetrahedron. It is computed using equation (3*a*) given initially or previously known charge density $\rho_\textrm{s}(\mathbf{r})$ on all faces. At the first and only at the first step, $\rho_\textrm{s}(\mathbf{r}) = 0$ and, therefore, $\mathbf{H}^\textrm{s}(\mathbf{r}) = 0$.
(c) Local permeability $\mu(|\mathbf{H}|)$ is then computed for every tetrahedron given $\mathbf{H} = \mathbf{H}^\textrm{p}+\mathbf{H}^\textrm{s}$ and the known *B*-*H* curve.
(d) Resulting differential contrast $K = (\mu^+-\mu^-)/(\mu^++\mu^-)$ is next computed for every face, either inner or on the boundary. In the last case, there is only one adjacent tetrahedron and $\mu^- = \mu_0$.
(e) Integral equation (3*c*) is finally solved next via the FMM with this particular set of contrasts *K*. This gives us the new magnetic charge densities $\rho_\textrm{s}(\mathbf{r})$ for every facet.
(f) The process repeats itself starting with step #2.
(g) The process stops when variations in the local permeability, $\mu(|\mathbf{H}|)$, and in the field within the core become small enough, i.e. the solution converges.

This successive substitution method converges fairly well (see section 3.2 for performance). At the same time, it requires accurate values of the neighbor potential integrals present in equations (3(a) and (c)). These values are precomputed as described in the prior studies [30, 32, 33].

When dealing with stronger nonlinearities (steeper *B*–*H* curves and/or complicated core geometry), smoothing of the local permeability for every tetrahedron at step #3 above might be done in the form

\begin{align*} \mu_\textrm{r}(\mathbf{r}_0) \rightarrow \alpha \mu_\textrm{r}(\mathbf{r}_0)+\frac{1}{4}(1-\alpha) \sum_{i=1}^4\mu_\textrm{r}(\mathbf{r}_i), \quad\alpha \leqslant 1 \end{align*}

where the summation is performed over four neighbor tetrahedra. For boundary tetrahedra, the summation is running over a smaller number of neighbors (three or two or one).

### 2.3. Solving the total field of the coil–core combination

It is important to emphasize that the coil–core combination is solved only once and up front, i.e. before using the head model. Two parameters required are the strength of the terminal coil current, *I*_0_, and the current change rate, $\mathrm{d} I/\mathrm{d} t$. The second parameter is purely linear; it could be altered *post factum* and at any step of the solution when desired.

The solution for the coil with the core is further utilized in the main TMS field computations. The coil–core configuration can be moved or rotated as required, without the need of recalculating the core magnetization. The coil+core solver’s output is as follows:

(a) Local permeability $\mu(|\mathbf{H}(\mathbf{r})|)$ and the total field $\mathbf{H}(\mathbf{r})$ within the core. These two are used to calculate the major parameter of interest—magnetization $\mathbf{M}(\mathbf{r})$ within the core from equation (2*b*)—as well as the inductance change.
(b) To alter inductance, we compute the extra energy added by the core and given by [45, 46]
  \begin{equation*} U{\,^\textrm{s}} = \frac{1}{2}\int_V \mathbf{M}(\mathbf{r}) \cdot \mathbf{B}^\textrm{p}(\mathbf{r}) \mathrm{d}\mathbf{r}.\end{equation*}
  The corresponding inductance correction $L^\textrm{s} = 2U^\textrm{s}/I_0^2$ is added to equation (5*b*) so that the total inductance with the core now becomes
  \begin{equation*} L = L^\textrm{p} + L^\textrm{s} = 2\frac{U^\textrm{p}}{I_0^2} + 2\frac{U^\textrm{s}}{I_0^2}. \end{equation*}
(c) The secondary vector potential $\mathbf{A}^\textrm{s}(\mathbf{r})$ anywhere in space is found from equation (2*c*) since the core magnetization is already known. Equation (1*b*) is thus modified by
  \begin{equation*} \mathbf{E}=\mathbf{E}^\textrm{p}+\mathbf{E}^\textrm{s}=-\frac{\mathrm{d} I}{\mathrm{d} t}\left[\mathbf{A}^\textrm{p}(\mathbf{r})+\mathbf{A}^\textrm{s}(\mathbf{r})\right] \end{equation*}
  for the combined induced electric field **E**. We prefer to store $\mathbf{M}(\mathbf{r})$ and then compute **E** from equation (4*c*) for any coil–head geometry as required. Following the established TMS computations terminology, this will be the primary or incident electric field of the coil with the magnetic core.
(d) If necessary, the secondary field $\mathbf{H}^\textrm{s}(\mathbf{r})$ outside the core is found from equation (3*c*) extended to the nonlinear core as described above. We prefer to store magnetic charges within the core and then compute $\mathbf{H} = \mathbf{H}^\textrm{p}+\mathbf{H}^\textrm{s}$ for any head geometry as required.

### 2.4. Calculation of coil inductance without the core

The magnetic energy of the primary coil field without the core is given by

\begin{equation*} U^\textrm{p} = \frac{1}{2} \int_V \mathbf{j}(\mathbf{r})\cdot \mathbf{A}^\textrm{p}(\mathbf{r})\mathrm{d}\mathbf{r}. \end{equation*}

The inductance of the primary coil, $L^\textrm{p}$, is found directly from the energy relation, $U^\textrm{p} = L^\textrm{p} I_0^2/2$, where *I*_0_ is the total terminal coil current. When the coils is discretized into *N* short straight line segments each carrying current *i*_*m*_ and located at **r**_*m*_, the coil inductance without the core is given by the Neumann formula (see, for example, [47–49])

\begin{equation*} L^\textrm{p}=2\frac{U^\textrm{p}}{I_0^2} = \frac{\mu_0}{4\pi} \sum_{m=1}^N \left|i_m s_m\cdot \sum_{n=1}^N \frac{i_n s_n}{\left|\mathbf{r}_m-\mathbf{r}_n\right|}\right|, \end{equation*}

where *s*_*m*_ and *s*_*n*_ are the lengths of the segments; *m* and *n* are the outer and inner summation indexes, respectively. The inner sum in equation (5*b*) is computed via the FMM [42, 43], as a potential of a single layer repeated *three times*. Those computations are done in parallel. After that, the outer sum is found directly. The *m* = *n* terms are set to zero, which is justified when the number of subdivisions, *N*, is large. We found that for precise inductance calculations via the Neumann formula, the ratio of average segment length to average segment spacing should be no less than 1–3.

### 2.5. Magnetic material models

Here, we consider an isotropic material with relative permeability *µ*_r_. Its most basic characteristic is the *anhysteretic* magnetization *B*–*H* curve, $\mu_\textrm{r} = \mu_\textrm{r}(|\mathbf{H}|)$, $\mathbf{B} = \mu_0\mu_\textrm{r}(|\mathbf{H}|)\mathbf{H}$ [17]—the key element of modeling magnetic hysteresis loops. It is fitted using the outermost *B*–*H* hysteresis loop. The anhysteretic curve strongly depends on the frequency of the sinusoidal (or pulsed) core test system (see, for example, [8]). The rest of the core properties might be accounted for as the hysteresis loss, classical eddy current loss, and anomalous loss [50].

Although it is possible to introduce hysteresis models of ferromagnetic materials into the numerical analysis, it is still relevant to use the single-valued *B*–*H* curve in such an analysis [39]. For the magnetically soft materials used in the TMS design, the hysteresis loops are relatively narrow, that is, the coersive force is smaller compared to ‘hard’ ferromagnetic material (see [39] for more in depth discussion). Therefore, the single-valued *B*–*H* curve adequately characterizes such materials in many applications [39]. In such a case, the losses are usually estimated using analytical formulae, while the nonlinear problem is solved using the Newton–Raphson or successive substitution method [39, 51–53].

In this study, only the single anhysteretic curve will be modeled. The core loss could be included into consideration *post factum*. Along with more sophisticated fitting models [39, 54], two popular models for the anhysteretic curve are the inverse-tangent model:

\begin{equation*} \mu_0 \mu_\textrm{r}(|\mathbf{H}|) = \frac{a_1 \tan^{-1}(a_2 |\mathbf{H}|)}{|\mathbf{H}|}+\mu_0,\end{equation*}

and the Froelich’s equation

\begin{equation*} \mu_0 \mu_\textrm{r}(|\mathbf{H}|) = \frac{1}{a_1+a_2|\mathbf{H}|}+\mu_0.\end{equation*}

Here, we adopt the forms of these constitutive relations as presented in [55], along with the limiting saturation value of *µ*_0_ on the right-hand sides of both equations. The presence of this *µ*_0_ term is sometimes neglected, but it is physically justified and is critical for the accurate numerical analysis at the high flux densities. In equation (6*a*), constant *a*_1_ has the units of T, while in equation (6*b*) constant *a*_1_ has the units of m H^−1^. In equation (6*a*), constant *a*_2_ has the units of m A^−1^, while in equation (6*b*) constant *a*_2_ has the units of 1/T.

**Figure 1.**
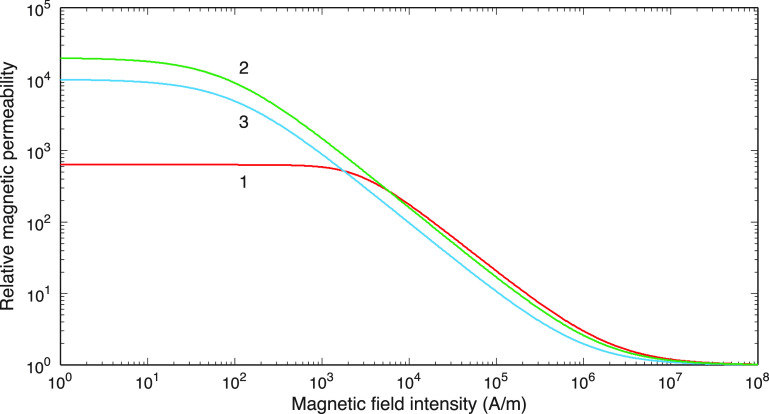
Three anhysteretic curves $\mu_\textrm{r} (|\mathbf{H}|)$ used in this study. 1 (red): a generic curve given by equation (6*a*) with $a_1 = 5/\pi\,{\textrm{T}}$, $a_2 = 5\times 10^{-4}$ m A^−1^. 2 (green): approximation of the M3 silicon steel datasheet of Arnold Magnetic Technologies™ at 2 kHz using equation (6*b*) with $a_1 = 40$ m H^−1^, $a_2 = 0.50$ T^−1^. 3 (blue): Approximation of the METGLAS® 2605-SA1 foil core datasheet described by equation (6*b*) with $a_1 = 80$ m H^−1^, $a_2 = 0.82$ T^−1^.

We will test three magnetic materials with gradually decreasing saturation fields shown in figure 1.

(a) The first curve in figure 1 corresponds to some generic nonlinear material with a high saturation field above 2 T as well as with a modestly varying permeability and a relatively small magnitude of $\mu_0 \mathrm{d}\mu_\textrm{r}/\mathrm{d}|\mathbf{H}|$. It is described by equation (6*a*) with $a_1 = 5/\pi\,{\textrm{T}}$, $a_2 = 5\times 10^{-4}$ m A^−1^.
(b) The second curve in figure 1 is an approximate curve fitting for a 2-mil M3 silicon steel core datasheet of Arnold Magnetic Technologies at 2 kHz [56]. This laminated core saturates at approximately 1.7 T. To obtain the single valued anhysteretic *B*–*H* curve, we use locus points of 6 symmetric hysteresis cycles. They are tabulated in the datasheet as the $(H_\textrm{max}, B_\textrm{max})$ pairs. The pair $(0,0)$ is added at the origin of the *B*–*H* plane. The entire curve is described by equation (6*a*) with $a_1 = 1.5\,{\textrm{T}}$, $a_2 = 1\times 10^{-2}$ m A^−1^ or by equation (6*b*) with $a_1 = 40$ m H^−1^, $a_2 = 0.50$ T^−1^ although some discrepancy appears between either analytical approximation and experiment below 300 A m^−1^. Namely, the curve supplied by the manufacturer exhibits a peak around 60 A m^−1^. Unfortunately, most of the simple approximations, including the inverse tangent or Froelich’s equation, lack to accurately model this peak. In many cases, this is not a problem if we are interested in modeling for *higher* magnetic flux densities [57], which is exactly the TMS case.
(c) The third curve in figure 1 is an accurate curve fitting data for a METGLAS® 2605-SA1 amorphous foil core datasheet [8]. This laminated core saturates at approximately $1.2\,{\textrm{T}}$. High-frequency losses of this material are low [8]. The *B*–*H* curve is now given by equation (6*b*) with $a_1 = 80$ m H^−1^, $a_2 = 0.82$ T^−1^.

All three material curves described above are characterized by quite different values of the maximum permeability variation rate, $\max\left(\left|\mu_0 \mathrm{d}\mu_\textrm{r}/\mathrm{d}|\mathbf{H}|\right|\right)$. This rate is important for stability considerations of the nonlinear numerical solution. It is known that at very high permeability variation rates, the nonlinear iterative solutions may easily diverge. For the first material, the maximum of the permeability variation rate is 0.11 *µ*H A^−1^, for the second one it is 304 *µ*H A^−1^, and it is equal to 126 *µ*H A^−1^ for the third material.

## 3. Results

### 3.1. Wire and core models in the software package

#### 3.1.1. Wire model

Computation of integrals (1) for any metal coil is straightforward. It is performed as described in [29] and supplement of [30] using the fast multipole method [42, 43]. All volumetric conductors are replaced by a computational wire grid consisting of a large number of straight, short, infinitely-thin filaments of electric current or segments. The number of these elementary filaments may easily exceed 100 000–1000 000 depending on the required solution accuracy. Then, all integrals (1) are discretized on filaments and are computed numerically using the fast multipole method or FMM [42, 43].

**Figure 2.**
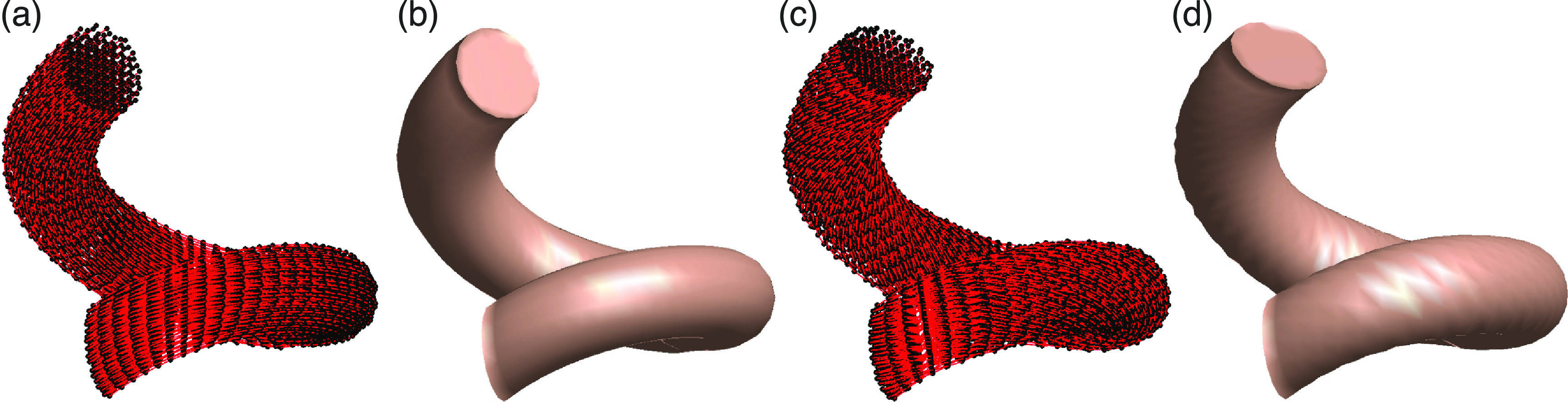
One turn of a helical spiral coil made of Litz wire. (a) Underlying computational wire grid with 9200 current filaments. (b) Rendered outward appearance of (a) in computer software. (c) The same but for a twisted Litz wire. Notice the twisting in the wire grid that is absent in (a); the twisting increases inductance. (d) Rendered outward appearance of (c) in the software; the surface rendering is less smooth compared to (b).

This approach is rather fast and flexible (typical run times are less than 1 s). It allows us to model a uniform current flow (the Litz wire), the skin layer effect in a solid copper conductor (current distribution close to the surface) as well as a twisted Litz wire which is encountered in some applications as shown in figure 2.

#### 3.1.2. Core model

**Figure 3.**
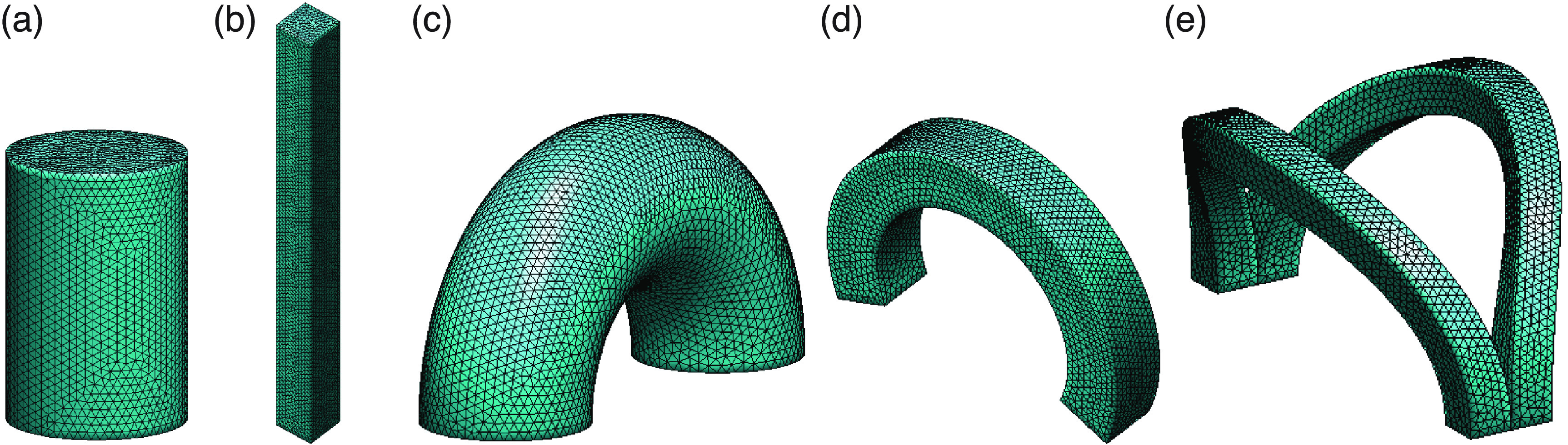
Internal tetrahedral mesh generator for simple core shapes and their combinations. Cylinder (a), rectangular cuboid (b), various C-shapes (c), (d) and their combinations (e) are made available.

Any custom core CAD file in *.stl format can be imported into the present software using MATLAB’s native STL converters. At the same time, an internal tetrahedral mesh generator is made available for simple (deformed or not) shapes such as cylinder (figure 3(a)), rectangular cuboid (figure 3(b)), C-shapes (figures 3(c) and (d)) and their combinations (see figure 3(e)), with an arbitrary volumetric (tetrahedral) and surface mesh resolution.

A number of validation examples have been constructed and tested which compare the present method and its accuracy for linear and nonlinear cores. Those include comparisons with the analytical formulae (linear inductance) [45, 48, 58], with a high-end commercial FEM magnetostatic solver ANSYS Maxwell, a part of ANSYS® Electronics Desktop 2021/R2 (nonlinear/linear fields, saturation/linear inductance), and with realistic TMS coil experiments (linear inductance).

All examples are contained in the ready-to-use downloadable software package as separate projects. Concurrent ANSYS projects are also included. Below, we present the most interesting (and challenging) examples in our opinion. Other examples are described in the supplementary material. Section S2 in the supplementary material presents a succinct software description along with the running sequences. We use the accessible self-contained MATLAB platform under Windows.

### 3.2. Comparison with ANSYS Maxwell FEM for three different materials (saturated core)

**Figure 4.**
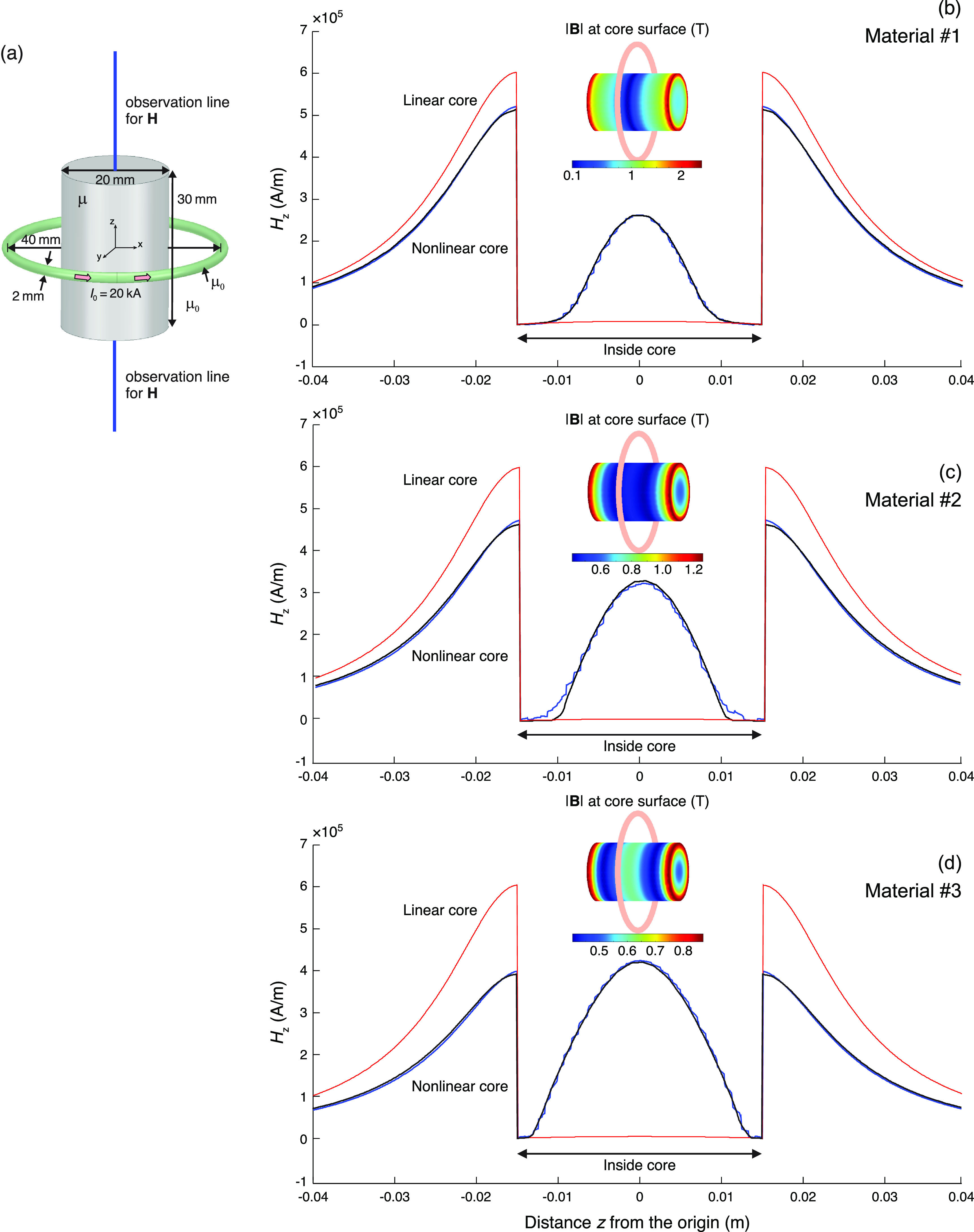
(a) Problem geometry and specific dimensions. (b)–(d) Magnetic field distributions along the core centerline for three different materials defined in figure 1. Blue curves: BEM-FMM results; black curves: ANSYS Maxwell results; thin red curves: the linear-core constant-permittivity solution.

The problem geometry is shown in figure 4(a). A simple solid cylindrical core is symmetrically located within a one-turn coil. The core mesh has ∼80 000 tetrahedra, the coil wire grid has ∼50 000 elementary current segments. Specific dimensions along with the total applied current and the observation line for **H** are shown in figure 4(a). The coil conductor is assumed to be non-magnetic and with a uniform electric current distribution across its cross-section (Litz wire). The homogeneous core is assumed to be non-conducting.

Three core materials #1, 2 and 3 from section 2.5 (shown in figure 1) with the gradually decreasing saturation of the magnetic flux within the core have been used. To assure the non-linear region of operation and core saturation in every case, the coil current is chosen as $I_0 = 20$ kA.

Two convergence measures of the non-linear successive substitution solution are the relative deviation in the magnetic charge density, *e*_1_, for all faces and the relative deviation in the permeabilities at tetrahedra centers for all tetrahedra, *e*_2_, i.e.

\begin{align*} e_1 &amp;= \|\rho_\textrm{s}^n-\rho_\textrm{s}^{n-1}\|/\|\rho_\textrm{s}^n\|, \end{align*}

\begin{align*} e_2 &amp;= \|\mu_\textrm{r}^n-\mu_\textrm{r}^{n-1}\|/\|\mu_\textrm{r}^n\|, \end{align*}

at every nonlinear iteration step *n*. Here, $\|\cdot\|$ is the Euclidean norm of the spatial distribution. Equation (7) is the relative deviation of the spatial field calculated as the norm over all surfaces or the norm over all volumetric elements.

In all three cases, a good monotonic convergence has been observed. For material #1, we reach the following values: $e_1 = 10^{-3}$, $e_2 = 5\times 10^{-3}$ after 20 iterations. For material #2, we reach $e_1 = 10^{-5}$, $e_2 = 5\times10^{-3}$ after 20 iterations, too. For material #3, we reach $e_1 = 10^{-3}$, $e_2 = 6\times10^{-3}$ after 25 iterations. The resulting magnetic fields along the core centerline are shown in figures 4(b)–(d) by blue curves. The corresponding ANSYS FEM solutions (with ∼2.2 M tetrahedra) are given by black curves. For comparison purposes, the linear-core solutions with $\mu_{\textrm{r}0} = \mu_\textrm{r} (|\mathbf{H}|\rightarrow 0) = 634$ (material #1), $\mu_{\textrm{r}0} = 19\,900$ (material #2, Froelich’s model), and $\mu_{\textrm{r}0} = 9950$ (material #3) are given by thin red curves. The ANSYS Maxwell FEM solutions execute in about 1.5–2 h using a 2.8 GHz multicore workstation (Windows platform) while the BEM-FMM solutions execute in 2–3 min on the same workstation.

Table 1 compares static inductance values obtained using both the numerical methods for the small-signal inductance values for the linear core and the inductance values for the saturated core at the given coil current of 20 kA. We emphasize that for the present (loose) coil winding the coil–core inductance is not very significantly affected by the magnetic core, either saturated or not. This fact was also noticed previously [10]. This is in stark contrast to the tight windings around the core considered, for instance, in the next example.

**Table 1.** Small-signal inductances and static inductances, for three saturated cores. Note a weak dependence of the overall inductance on the magnetic core for the loose core-coil configuration in figure 4(a).

	Linear core	Nonlinear core	Average core *µ*_r_
Inductance, nH	$I_0=1$ mA	$I_0=20$ kA	$I_0=20$ kA
BEM-FMM material 1	115	107	75
BEM-FMM material 2	116	103	29
BEM-FMM material 3	116	96	10
ANSYS material 1	116	107	NA
ANSYS material 2	116	103	NA
ANSYS material 3	116	96	NA

### 3.3. Comparison with measurements for a rat coil with and without magnetic core

#### 3.3.1. Coil construction

**Figure 5.**
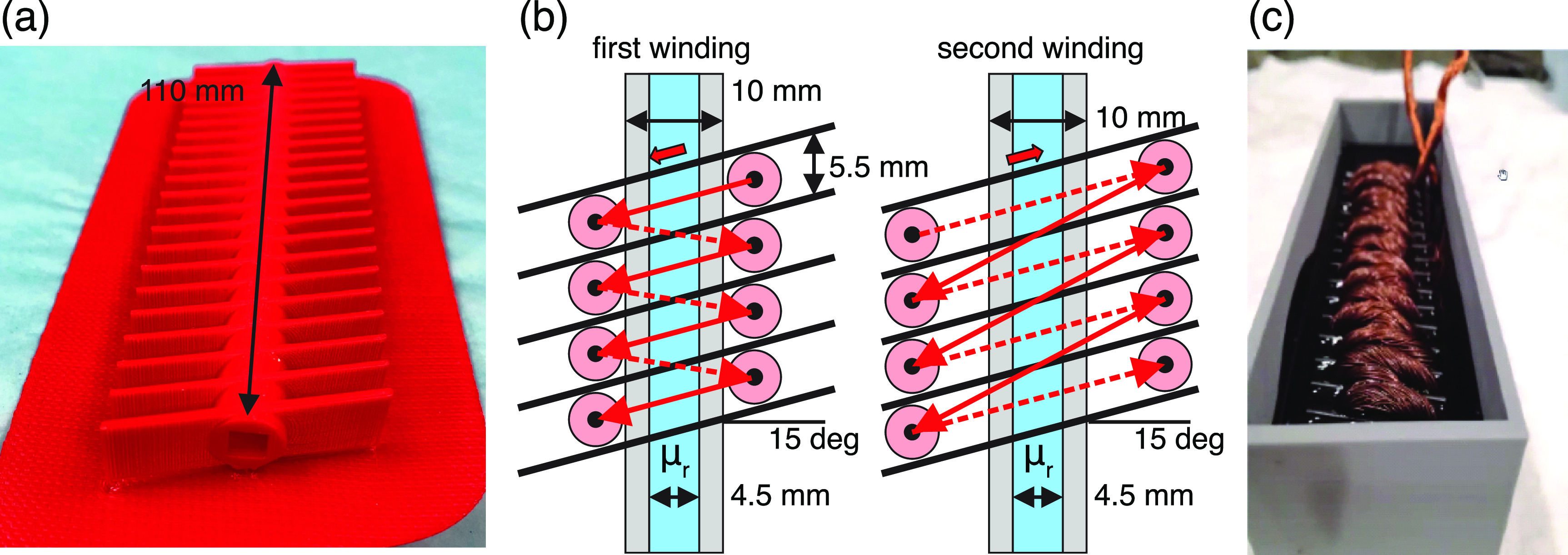
(a) Coil winding template. (b) Geometry dimensions for both windings. (c) Coil prototype before epoxy filling.

Figure 5 shows the geometry and major dimensions for a family of experimental focal TMS rat coils that were constructed. The coil windings, which are based on a plastic template with a 15^∘^ skew angle shown in figure 5(a), consist of two (or more) helical spirals of a variable pitch shown in figure 5(b). For the particular design considered, every spiral has 20 turns and is made of a custom Litz wire. The total coil length of the coil is 110 mm.

The single-wire radius in the Litz bundle is 0.15 mm (29 gauge with insulation), the number of single wires in the Litz bundle is 100. According to the packing tables [59], the optimal dimensionless radius of the circles in the container circle is 0.0902 in this case given that the latter has the radius of one. This yields the optimal radius of the bundle being equal to 1.663 mm. This is the best possible estimate; a more realistic estimate for the bundle radius used here is approximately 2 mm. This gives us the bundle diameter of the coil conductor of approximately 4 mm. Additionally, Litz wire twisting was estimated and then used in the model construction below.

A rectangular laminated magnetic core is tightly inserted in the rectangular opening of the template shown in figure 5(a). The core consists of multiple 2-mil sheets of M3 grain oriented silicon steel (Arnold Magnetic Technologies, Rochester, NY, USA), which is material #2 of the previous example.

#### 3.3.2. Modeling

**Figure 6.**
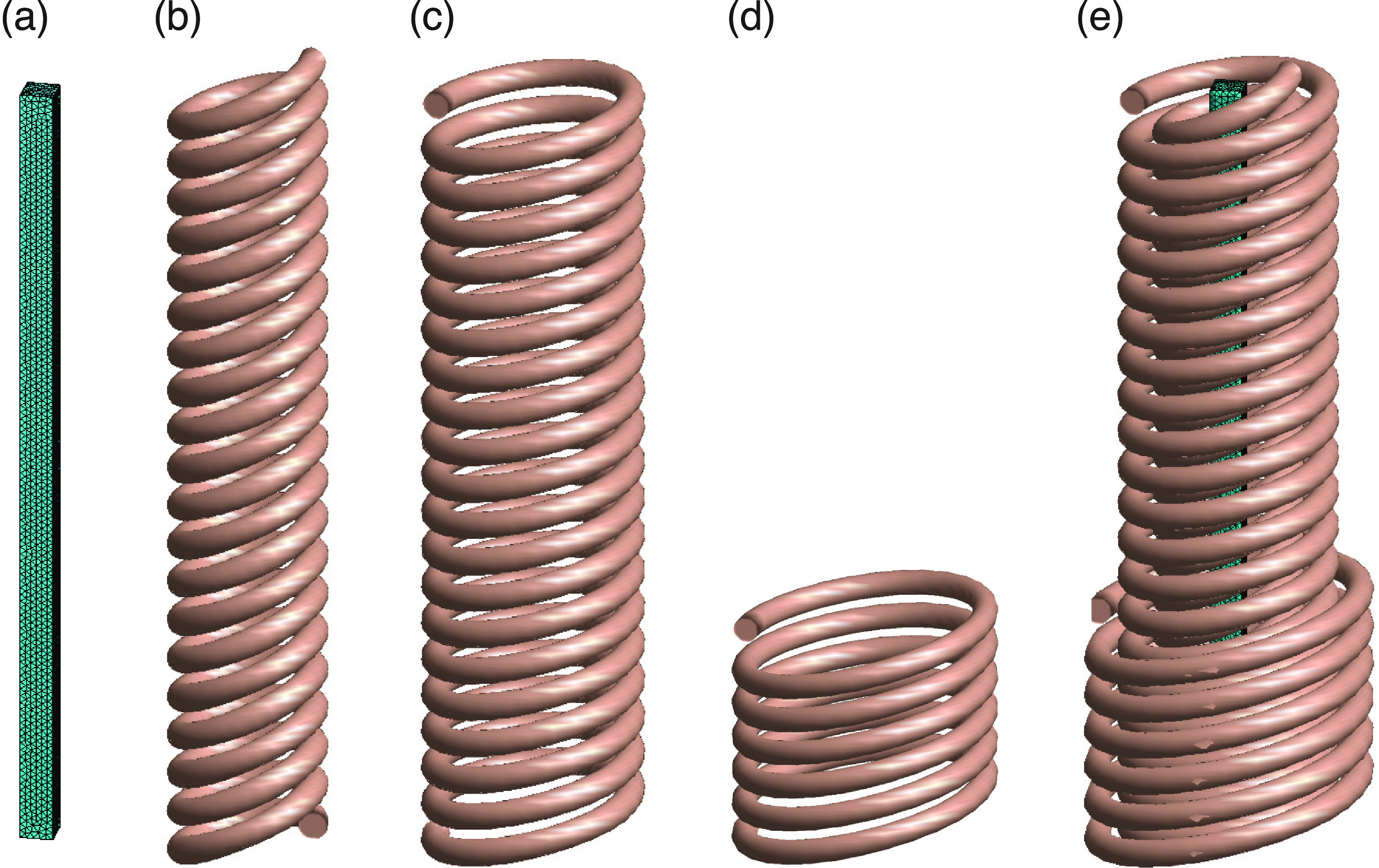
Computational model of the TMS rat coil. (a) The long square-bar magnetic core of M3 silicon steel. (b) Skewed inner helical spiral with the radius of 9 mm and a variable pitch. (c) Similar outer spiral with the radius of 16 mm. (d) A complete 20+20+6 coil assembly (the 20+20 assembly looks similar). Litz wires have been numerically ‘twisted’ as shown in figure 2.

The computational model consists of several components shown in figure 6: (a) the long rectangular core of M3 silicon steel, (b) the skewed inner helical spiral with the radius of 9 mm and a variable pitch, (c) the similar outer spiral with the radius of 16 mm rotated by 180^∘^ and, (d) an optional 6-turn third helical spiral used to boost the injected electric field.

The underlying computational mesh includes approximately 200 000 elementary current segments modeling metal windings and approximately 25 000 tetrahedra modeling the magnetic core. Small-signal (linear-core) simulations including inductance and *H*- as well as *E*-field calculations run in approximately 1–2 s on a 2.8 GHz computer. For the latter case, the magnetization in the entire core volume is computed as described above.

#### 3.3.3. Small-signal coil inductances

Table 2 summarizes the small-signal inductances of the two coils (20+20 and 20+20+6 turns), which were measured using an LCR meter (model: Keysight U1732C) at 1 kHz. The small-signal relative magnetic permeability of the core was assumed to be approximately 19 900 following the Froelich’s curve from figure 1 for the M3 silicon steel (material #2). The agreement between experiment and modeling is within 10% for all reported values. Small variations in the coils’ assembly have been tested and nearly identical inductance values were obtained.

**Table 2.** Comparison between measured and modeled inductances for two different experimental rat TMS coils with and without the magnetic core. M3 Silicon steel (material #2) was modeled.

	Coil A: 2 layers of windings		Coil B: 3 layers of windings	
	20+20 turns		20+20+6 turns	
Inductance, *µ*H	No core	With core	No core	With core
Measured	7.0	40.0	8.5	50.0
Modeled	7.0	37.0	8.4	47.8

#### 3.3.4. Static inductance as a function of increasing coil current

The initial (small-signal) static inductance of the coil with the core decreases when the current increases to typical TMS levels and the core saturates. This important tendency is illustrated in table 3 for the experimental TMS rat coil with 20+20+6 turns and with the square M3 magnetic core from figure 6. Realistic TMS coil current strengths were considered.

**Table 3.** Static inductance and relative permeability as function of coil current. M3 Silicon steel (material #2) was modeled for the 20+20+6 experimental rat coil.

Coil current, A	100	1000	3000
Modeled inductance, *µ*H	27.9	10.3	9.0
Average core *µ*_r_	99.5	7.6	2.9

## 4. Discussion

### 4.1. Method convergence

It is known that the nonlinear FEM solvers for the magnetic cores do not necessarily converge. For example, the COMSOL application notes specifically discuss the cases where the convergence is absent [60]. The present method will also not converge for an arbitrary *B*–*H* curve. The critical parameter introduced in section 2.5 is the permeability variation rate, $|(\mathrm{d}\mu/\mathrm{d}|\mathbf{H}|)|$, of a magnetic material. If this rate is very high (the nonlinearity is very strong), the converge cannot be guaranteed. Several methods have been tested including local permeability averaging as in equation (3*d*), Jacobian smoothing of magnetic contrast variations at every iteration step as well as introducing more integration points over the tetrahedra volumes and the following averaging. None of these methods generated ultimately better results.

The only method which was converging in all tested cases is that which computed the averaged *H*-field value within a tetrahedron based on four field values just inside its four faces at the face centers i.e. directly via the surface magnetic charges. However, this method generated choppy fields within the core and was less favorably compared to the FEM solver ANSYS Maxwell within the core. Therefore, it was not implemented, but was rather used as a small auxiliary contribution to assure the convergence in the demanding cases.

At present, we could probably guarantee the method convergence for typical TMS field strengths and for the simple analytical *B*–*H* approximation curves such as equations (6), and more accurate yet *smooth* analytical interpolations [8, 50, 54, 55]. Note again that this could be quite enough if we are interested in modeling for higher magnetic flux densities [57], which is exactly the TMS case.

### 4.2. Model limitations

Only the simple anhysteretic magnetization curve has been modeled in the present study, without including the core losses described in the introduction into consideration. The core losses may be significant for many magnetic materials and at shorter pulse durations. Their accurate description will require a separate detailed study.

No material anisotropy has been included although its modeling is rather trivial with the suggested method. At step 3 in section 2.2.2, the local permeability $\mu(|\mathbf{H}|)$ for every tetrahedron could be different in different directions. This can be taken into account by adjusting the magnetic contrast for the four faces of the tetrahedron using the product of a permeability tensor and the normal vector of every facet. Unfortunately, the FEM solver ANSYS Maxwell cannot be used for comparison purposes in this case: it can either model the *B*–*H* curve or the anisotropy of an otherwise linear magnetic material.

### 4.3. Temporal considerations

Although the our solver is quasistatic in nature, there are temporal waveform issues to consider. For conventional TMS sinusoidal pulses, the coil current and electric field waveforms are 180^∘^ out of phase. Consequently, when the coil current reaches its maximum—when we expect higher effects from the nonlinear core, possibly deeper core saturation—the induced electric field is zero at this time. Typically, one is interested in the electric field distribution at its peak as it is thought to reflect maximum impact on neuronal activation. In other devices, such as the controllable pulse width stimulator [61], peak coil current is reached when the electric field is still quite high, albeit not at maximum. Interestingly, the induced change in neuronal membrane potential reaches maximum at peak coil current [61]. Depending on the outcome of interest, one might have to perform multiple simulations at different time points along the current waveform. In any case, our model requires both the coil current and its time derivative values as input parameters. The effect of the magnetic core on electric field and neuronal activation can be studied at different time points along any arbitrary pulse waveform.

## 5. Conclusions

The present study reports the simple, accurate, and accessible algorithm for modeling TMS coils with a (nonlinear) magnetic core and validates the algorithm through comparison with FEM simulations and experiments. The algorithm is using the BEM-FMM applied to all facets of the tetrahedral core mesh as a single-state solution and the successive substitution method to assure the nonlinear convergence of the subsequent states. The coil–core combination is solved only once, before incorporating the head model. The resulting primary TMS electric field is proportional to the total magnetic vector potential in the quasistatic approximation; it therefore also employs the precomputed core magnetization. Changes in the regular TMS computation pipeline are reduced to a minimum. The method demonstrates excellent convergence for typical TMS field strengths and for analytical *B*–*H* approximations of the experimental magnetization curves in the form of Froelich’s equation or the inverse tangent equation. Average execution times have been between 1 and 3 min on a common multicore workstation. The method also outputs coil’s self- or mutual inductances, with or without the magnetic core.

Finally, we provided numerous example applications as part of our codebase, including models for rodent and human TMS coils with a magnetic core. These examples have potential impact on translational and clinical applications of TMS, as well as future technology development for TMS.

## Data Availability

The data that support the findings of this study are openly available at the following URL/DOI: https://tinyurl.com/rdkdrkck.

## Code Availability

The ready-to-use software package is available at https://tinyurl.com/rdkdrkck. It contains eight validation and application examples in the form of separate projects. Concurrent ANSYS Maxwell projects are also included. We employ the accessible MATLAB platform under Windows.

## References

[1] Chen C-W (2013). Magnetism and Metallurgy of Soft Magnetic Materials.

[2] Epstein C M, Davey K R (2002). Apparatus and method for transcranial magnetic brain stimulation,
including the treatment of depression and the localization and characterization of
speech arrest. US Patent.

[3] Davey K R, Epstein C M (2002). Magnetic nerve stimulation seat device. US Patent.

[4] Goetz S M, Deng Z-D (2017). The development and modeling of devices and paradigms for transcranial
magnetic stimulation. Int. Rev. Psychiatry.

[5] Neuronetics, Inc. (2019). News release details. https://ir.neuronetics.com/news-releases/news-release-details/neuronetics-inc-celebrates-dr-charles-epsteins-contributions.

[6] Leary A M, Ohodnicki P R, McHenry M E (2012). Soft magnetic materials in high-frequency, high-power conversion
applications. JOM.

[7] Sarker P C, Islam M R, Guo Y, Zhu J, Lu H Y (2019). State-of-the-art technologies for development of high frequency
transformers with advanced magnetic materials. IEEE Trans. Appl. Supercond..

[8] U.S. Department of Energy—National Energy Technology
Laboratory (2018). METGLAS® 2605-SA1 core datasheet. https://www.netl.doe.gov/sites/default/files/netl-file/METGLAS-2605-SA1-Core-Datasheet_approved.

[9] Ouyang G, Chen X, Liang Y, Macziewski C, Cui J (2019). Review of Fe-6.5 wt%Si high silicon steel—a promising soft magnetic
material for sub-kHz application. J. Magn. Magn. Mater..

[10] Salvador R, Miranda P C, Roth Y, Zangen A (2009). High permeability cores to optimize the stimulation of deeply located
brain regions using transcranial magnetic stimulation. Phys. Med. Biol..

[11] Carmona I C, Kumbhare D, Baron M S, Hadimani R L (2021). Quintuple AISI 1010 carbon steel core coil for highly focused
transcranial magnetic stimulation in small animals. AIP Adv..

[12] RamRakhyani A K, Lazzi G (2013). Analysis of non-linear magnetic core for magnetic neural
stimulators.

[13] RamRakhyani A K, Lazzi G (2014). Ferrite core non-linearity in coils for magnetic
neurostimulation. Healthc. Technol. Lett..

[14] Meng Q, Jing L, Badjo J P, Du X, Hong E, Yang Y, Lu H, Choa F-S (2018). A novel transcranial magnetic stimulator for focal stimulation of
rodent brain. Brain Stimul..

[15] Wilson M T, Tang A D, Iyer K, McKee H, Waas J, Rodger J (2018). The challenges of producing effective small coils for transcranial
magnetic stimulation of mice. Biomed. Phys. Eng. Express.

[16] Khokhar F A, Voss L J, Steyn-Ross D A, Wilson M T (2021). Design and demonstration *in vitro* of a
mouse-specific transcranial magnetic stimulation coil. IEEE Trans. Magn..

[17] Deng Z-D, Lisanby S H, Peterchev A V (2014). Coil design considerations for deep transcranial magnetic
stimulation. Clin. Neurophysiol..

[18] Koponen L M, Nieminen J O, Mutanen T P, Stenroos M, Ilmoniemi R J (2017). Coil optimisation for transcranial mangetic stimulation in realistic
head geometry. Brain Stimul..

[19] Yoon H, Kim I, Shin P S, Koh C S (2011). Finite element implementation of a generalized Chua-type vector
hysteresis model and application to iron loss analysis of three-phase
transformer. IEEE Trans. Magn..

[20] Wang X, Xie D, Bai B, Takahashi N, Yang S (2008). 3-D FEM analysis in electromagnetic system considering vector
hysteresis and anisotropy. IEEE Trans. Magn..

[21] Leonard P J, Rodger D, Karagular T, Coles P C (1995). Finite element modelling of magnetic hysteresis. IEEE Trans. Magn..

[22] Miano G, Serpico C, Verolino L, Visone C (1995). Comparison of different hysteresis models in FE analysis of magnetic
field diffusion. IEEE Trans. Magn..

[23] Hoffmann K, Bastos J P A, Leite J V, Sadowski N, Barbosa F (2017). A vector Jiles–Atherton model for improving the FEM
convergence. IEEE Trans. Magn..

[24] Li W, Kim I H, Jang S M, Koh C S (2011). Hysteresis modeling for electrical steel sheets using improved vector
Jiles–Atherton hysteresis model. IEEE Trans. Magn..

[25] Benabou A, Clénet S, Piriou F (2003). Comparison of Preisach and Jiles–Atherton models to take into account
hysteresis phenomenon for finite element analysis. J. Magn. Magn. Mater..

[26] Saturnino G B, Puonti O, Nielsen J D, Antonenko D, Madsen K H, Thielscher A (2019). SimNIBS 2.1: a comprehensive pipeline for individualized electric
field modelling for transcranial brain stimulation. Brain and Human Body Modeling: Computational Human Modeling at Embc
2018.

[27] Saturnino G B, Madsen K H, Thielscher A (2019). Electric field simulations for transcranial brain stimulation using
FEM: an efficient implementation and error analysis. J. Neural Eng..

[28] Saturnino G B, Thielscher A, Madsen K H, Knosche T R, Weise K A (2019). A principled approach to conductivity uncertainty analysis in electric
field calculations. Neuroimage.

[29] Makarov S N, de Lara L I N, Noetscher G M, Nummenmaa A (2019). Modeling primary fields of TMS coils with the fast multiple
method. bioRxiv Preprint.

[30] Makarov S N, Wartman W A, Daneshzand M, Fujimoto K, Raij T, Nummenmaa A (2020). A software toolkit for TMS electric-field modeling with boundary
element fast multipole method: an efficient MATLAB implementation. J. Neural Eng..

[31] Makarov S N, Noetscher G M, Nazarian A (2015). Low-Frequency Electromagnetic Modeling for Electrical and Biological Systems
Using Matlab.

[32] Makarov S N, Golestanirad L, Wartman W A, Nguyen B T, Noetscher G M, Ahveninen J P, Fujimoto K, Weise K, Nummenmaa A R (2021). Boundary element fast multipole method for modeling electrical brain
stimulation with voltage and current electrodes. J. Neural Eng..

[33] Makarov S N, Hämäläinen M, Noetscher G M (2020). Boundary element fast multipole method for enhanced modeling of
neurophysiological recordings. IEEE Trans. Biomed. Eng..

[34] Van Bladel J G (2007). Electromagnetic Fields.

[35] Lean M, Wexler A (1982). Accurate field computation with the boundary element
method. IEEE Trans. Magn..

[36] Rucker W M, Richter K R (1988). Three-dimensional magnetostatic field calculation using boundary
element method. IEEE Trans. Magn..

[37] Krstajic B, Andelic A, Milojkovic S, Babic S, Salon S (1992). Nonlinear 3D magnetostatic field calculation by the integral equation
method with surface and volume magnetic charges. IEEE Trans. Magn..

[38] Arrott A S, Templeton T L (2018). Using magnetic charge to understand soft-magnetic
materials. AIP Adv..

[39] Vladimirov V S (1971). Equations of Mathematical Physics.

[40] Saad Y (2003). Iterative Methods for Sparse Linear Systems.

[41] Oseledets V, Dolgov S, Kazeev V, Savostyanov D, Lebedeva O, Zhlobich P, Mach T, Song L (2016). TT-Toolbox. FGMRES.

[42] Greengard L, Rokhlin V (1987). A fast algorithm for particle simulations. J. Comput. Phys..

[43] Gimbutas Z, Greengard L, Magland J, Rachh M, Rokhlin V (2021). fmm3D Documentation. Release 0.1.0..

[44] Mayergoyz I, Chari M, D’Angelo J (1987). A new scalar potential formulation for three-dimensional magnetostatic
problems. IEEE Trans. Magn..

[45] Smythe W R (1950). Static and Dynamic Electricity.

[46] Jackson J D (1998). Classical Electrodynamics.

[47] Sonntag C L W, Lomonova E A, Duarte J L (2008). Implementation of the Neumann formula for calculating the mutual
inductance between planar PCB inductors.

[48] Dengler R (2016). Self inductance of a wire loop as a curve integral. Adv. Electromag..

[49] Liu S, Su J (2019). Accurate expressions of mutual inductance and their calculation of
Archimedean spiral coils. Energies.

[50] Sudhoff S D (2014). Power Magnetic Devices: A Multi-Objective Design Approach.

[51] Janicke L, Kost A (1998). Convergence properties of the Newton–Raphson method for nonlinear
problems. IEEE Trans. Magn..

[52] Das R, Lowther D A (2013). Acceleration of field computation involving HTS. IEEE Trans. Magn..

[53] Niu S, Fu W N, Ho S L (2015). Nonlinear convergence acceleration of magnetic field
computation. IEEE Trans. Magn..

[54] Shane G M, Sudhoff S D (2010). Refinements in anhysteretic characterization and permeability
modeling. IEEE Trans. Magn..

[55] Dadić M, Jurc̈ević M, Malarić R (2020). Approximation of the nonlinear B-H curve by complex exponential
series. IEEE Access.

[56] Arnold Magnetic Technologies (2012). 2 mil grain oriented silicon steel hysteresis curve at 2000
Hz. https://www.arnoldmagnetics.com/wp-content/uploads/2017/10/2-Mil-Grain-Oriented-Silicon-Steel-Hysteresis-Curve-at-2000Hz.pdf.

[57] Dadić M (2021).

[58] Grover F W (2009). Inductance Calculations.

[59] Specht E (2021). The best known packings of equal circles in a circle (complete up to
*N* = 2600). http://hydra.nat.uni-magdeburg.de/packing/cci/.

[60] COMSOL knowledge database improving convergence in nonlinear time dependent
models. https://www.comsol.com/support/knowledgebase/1127.

[61] Peterchev A V, Jalinous R, Lisanby S H (2008). A transcranial magnetic stimulator inducing near-rectangular pulses
with controllable pulse width (cTMS). IEEE Trans. Biomed. Eng..

